# Episiotomy in Southern Brazil: prevalence, trend, and associated factors

**DOI:** 10.11606/s1518-8787.2022056003908

**Published:** 2022-04-11

**Authors:** Juraci A. Cesar, Luana P. Marmitt, Raúl A. Mendoza-Sassi

**Affiliations:** I Universidade Federal do Rio Grande Faculdade de Medicina Programa de Pós-graduação em Saúde Pública Rio Grande RS Brasil Universidade Federal do Rio Grande. Faculdade de Medicina. Programa de Pós-graduação em Saúde Pública. Rio Grande, RS, Brasil; II Universidade do Oeste de Santa Catarina Programa de Pós-Graduação em Biociências e Saúde Flor da Serra SC Brasil Universidade do Oeste de Santa Catarina. Programa de Pós-Graduação em Biociências e Saúde. Flor da Serra, SC, Brasil

**Keywords:** Episiotomy, trends, Prevalence, Risk Factors, Socioeconomic Factors

## Abstract

**OBJECTIVE:**

To identify and analyze the prevalence, trend, and factors associated with episiotomy in Rio Grande, in the state of Rio Grande do Sul, Southern Brazil.

**METHODS:**

A single, standardized questionnaire was applied to all pregnant women, residents in the municipality of Rio Grande, who had children in local hospitals between January 1 and December 12 of the years 2007, 2010, 2013, 2016 e 2019. Demographic and socioeconomic characteristics were investigated, as well as the assistance received during pregnancy and delivery. Chi-square test was used to compare proportions and Poisson regression with robust variance adjustment was used for multivariable analysis. Prevalence ratio (PR) was used as effect measure.

**RESULTS:**

Among the 12,645 births that occurred in the five years, 5,714 (45.2%) were vaginal delivery. Of these mothers, 2,930 (51.3%; 95%CI: 50.0%–52.6%) underwent episiotomy. Over this period, the episiotomy rate decreased from 70.9% (68.4–73.5) in 2007 to 19.4% (17.1–21.7) in 2019. Adjusted analysis showed a high PR of episiotomy occurrence among women who were young (PR = 2.23; 95%CI: 1.89–2.63), had higher education (PR = 1.21; 95%Cl: 1.03–1.42), had a higher family income (PR = 1.25; 95%CI: 1.10–1.41), were primiparous (PR = 3.41; 95%CI: 2.95–3.95), had prenatal care in the private sector (PR = 1.25; 95%CI: 1.07–1.46), had oxytocin-induced labor (PR = 1.18; 95%CI:1.09–1.27), underwent forceps (PR = 1.32; 95%CI: 1.16–1.50), and whose newborn weighed 4,000 g or more (PR = 1.43; 95%CI: 1.14–1.80).

**CONCLUSION:**

Although the prevalence of episiotomy fell sharply within the studied period, its occurrence is more likely among women at lower risk of birth complications.

## INTRODUCTION

Episiotomy, or perineotomy, refers to the surgical incision designed to reduce the incidence of severe perineal tears during labor^1^. The two most common types are medial and mediolateral^2^. Usually, episiotomy is referred as a simple, quick, low-cost surgical procedure that avoids or reduces perineal lacerations, reduces maternal-fetal distress, and accelerates labor^1^.

Against its realization is the lack of scientific evidence of its benefits, primarily when employed routinely. Several studies have shown that episiotomy favors bleeding and injuries in the perineal region, promotes sphincter trauma, facilitates fecal incontinence and flatulence, and prolongs postpartum pain, among other complications^1^.

Notwithstanding, episiotomy is the second most commonly performed surgical procedure among women of childbearing age, losing out only to cesarean sections. Its prevalence varies worldwide – for example, Cambodia^5^reports 90%; the Netherlands^6^, 11%; France^7^, 20%; and Canada, 17%^8^. Its prevalence in Brazil ranges from 47% in public hospital to 68% in private hospital^9^. Its occurrence may be universal, depending on the professional performing the delivery, hospital, and maternal characteristics, especially if primiparous^4^.

Few population-based studies are found on the subject despite the high prevalence. The quantification of episiotomy at the population level, knowledge of the profile of those submitted to this procedure, and the identification of factors associated with its occurrence could contribute to its rational use. This could help reduce the incidence of severe perineal laceration due to poor indication of this procedure, especially where practice is routine^10^.

This study aims to identify and analyze the prevalence, trend, and factors associated with episiotomy among all puerperal women living in the municipality of Rio Grande, Southern Brazil, in 2007, 2010, 2013, 2016, and 2019.

## METHODS

This study includes all pregnant women who delivered between January 1 and December 31 of 2007, 2010, 2013, 2016, and 2019 in the only two local maternity hospitals in Rio Grande, Rio Grande do Sul State, Southern Brazil. This municipality is in the coastal stretch, 250 km from the border with Uruguay. In this period, the local population ranged from 195,000 to 211,000 inhabitants.

For mothers to be included in this study, their newborns had to weight at least 500 grams or have at least a 20 weeks gestational age. They also had to be residents in the urban or rural areas of the municipality. We employed a cross-sectional design, interviewing women in the maternity ward within 48 hours after delivery.

The data were collected through a single pre-coded and printed questionnaire in 2007–2013. In 2016–2019, an electronic version of the same questionnaire was used, with data entry on tablets. On this occasion, we employed the REDCap (Research Electronic Data Capture)^11^application, and the data were uploaded daily through an online platform to the Universidade Federal do Rio Grande (FURG). This questionnaire investigated the women’s demographic, reproductive history, lifestyle habits characteristics, socioeconomic status, housing and sanitation conditions of their families, the care received during pregnancy and delivery, and their access to, and use of, preventive and curative health services. Episiotomy was defined as a surgical incision in the perineal region at the time of delivery^1^.

Three interviewers were selected to work in the study. After training, a pilot study was carried out at the two local maternity wards in December before the onset of data collection. During the study period, interviewers visited the maternity wards daily. All of them worked in a monthly rotation system in order to work at both hospitals.

The identification of pregnant women - was based on the access to daily birth records and in visits to the new mothers in the maternity ward. The study’s objectives were explained to the parturient, who were residents of Rio Grande, as she was identified. If in agreement, they signed the consent form, and the interview was performed. At the end of each day, the interviewer coded the applied questionnaires and delivered them at the study headquarters, where the open-ended questions were coded; the questionnaires were reviewed and delivered for double entry by independent professionals. Entries were compared for each batch of 100 questionnaires. Data were entered with Epidata^12^, corrected using Epi Info 6.04^13^, and analyzed with Stata version 13.0. Further details on this methodology are provided in previous publication^14^.

The multivariable analysis was based on the hierarchical model shown in Box. The statistical significance of each variable in the model was evaluated using the Wald and linear trend tests. Initially, each block of variables of a given level was included in the hierarchical analysis model, keeping all variables with a p-value of ≤ 0.20. In this model, the variables located at a hierarchically higher level than that of the variable in question were considered potential confounders concerning the outcome, whereas variables located at lower levels were considered potential mediators. Chi-square test was used for comparisons between proportions and Poisson regression was used for the multivariable analysis.

**Box:** Hierarchical analysis model for factors associated with episiotomy in Rio Grande, Southern Brazil, 2007–2019.

Level	Variables	
I	Demographic: Skin color and age	Socioeconomic: Mother’s schooling and household monthly income
II	Reproductive: Parity	
III	Current pregnancy care: Performed antenatal care visits in public sector or private sector, trimester in which antenatal care started, number of consultations performed, weight gained during pregnancy, type of health services used during the delivery.	
IV	Labor delivery and nutritional status of newborn: induction of labor, use of forceps and birthweight	
Outcome	Occurrence of episiotomy	

Regarding quality control, about 10% of telephone or home visit interviews were redone using a questionnaire with critical questions. The kappa index was used to compare responses obtained with those of the interviewer. The agreement obtained by this index ranged from 0.61 (pregnancy planning) to 0.99 (the type of delivery), which is satisfactory.

## RESULTS

These five years recorded 12,914 births in this municipality. Of this total, 12,645 (98%) were successfully investigated. The analysis included only vaginal delivery (5,714 births, or 45.2% of the total).

Table 1 shows that 23% of all mothers were adolescents (< 20 years old); 65% were white; 52% had up to four years of schooling; 35% of them belonged to the lowest income quartile; 39% were primiparous; 72% started antenatal care visits in the first trimester of pregnancy and performed at least six medical visits; 60% had used oxytocin; 94% had delivery in the public sector; and 11% of all their children were born with low birth weight (< 2,500g). This same table shows that the prevalence of episiotomy was highest amongst adolescent women, white, with over nine schooling years, in the highest income quartile, primiparous, who started antenatal care in the first trimester, performed more than six medical visits, received oxytocin, were submitted to forceps, and had a newborn with a birth weight of 3,000-3,500g.

**Table 1:** Prevalence of episiotomy by category of the studied variable. Rio Grande, RS, Brazil, 2007–2019.

Variable	Prevalence of episiotomy (%)	Total of mothers (%)
Mother’s age (full years)		
11–19	66.1 (860)	22.8 (1,301)
20–24	52.6 (883)	29.4 (1,679)
25–29	49.0 (612)	21.9 (1,250)
30–34	43.1 (396)	16.1 (919)
≥ 35	31.7 (179)	9.9 (565)
Skin color		
White	52.6 (1,965)	64.9 (3,706)
Brown/mixed	49.8 (633)	22.2 (1,270)
Black	47.1 (332)	12.9 (738)
Mother’s schooling (full years)		
0–4	40.5 (201)	48.4 (2,765)
5–8	48.9 (1,199)	42.9 (2,453)
≥ 9	55.3 (1,530)	8.7 (496)
Household monthly income (quartiles) (n = 5,440)		
First (lowest)	48.1 (904)	34.6 (1,880)
Second	48.7 (728)	27.5 (1,495)
Third	55.0 (723)	24.2 (1,315)
Quarter (highest)	56.9 (427)	13.8 (750)
Parity		
1	71.1 (1,567)	38.6 (2,203)
2	51.1 (1,042)	35.7 (2,039)
≥ 3	21.8 (321)	25.8 (1,472)
Trimester of first antenatal care visit (n = 5,355)		
First	55.2 (2,116)	71.5 (3,831)
Second	47.5 (644)	25.3 (1,357)
Third	36.5 (106)	3.1 (167)
Number of antenatal care visits performed		
0 or 1	30.5 (126)	7.2 (413)
3–5	46.2 (556)	21.1 (1,203)
≥ 6	54.9 (2,248)	71.7 (4,098)
Induction of labor		
Yes	46.0 (1,563)	59.5 (3,401)
No	59.1 (1,367)	40.5 (2,313)
Type of health services used for delivery		
Public	50.1 (2,695)	94.2 (5,385)
Private	71.4 (235)	5.8 (329)
Use of forceps		
Yes	82.8 (275)	5.8 (332)
No	49.3 (2,655)	94.2 (5,382)
Birth weight (in grams) (n = 5,706)		
< 2,500	37.7 (237)	11.0 (629)
2,500–2,999	50.8 (701)	24.2 (1,379)
3,000–3,499	55.4 (1,273)	40.3 (2,299)
3,500–3,999	51.9 (601)	20.3 (1,159)
≥ 4,000	48.8 (117)	4.2 (240)
Prevalence of episiotomy	51.3 (2,930)	100.0 (5,714)

Figure shows that episiotomy rate dropped from 70.9% (95%CI: 68.4%–73.5%) in 2007 to 19.4% (95%CI: 17.1%–21.7%) in 2019. Within the period, 51% (95%CI: 50.0%–52.6%) were submitted to episiotomy (p trend < 0.001).

**Figure:**
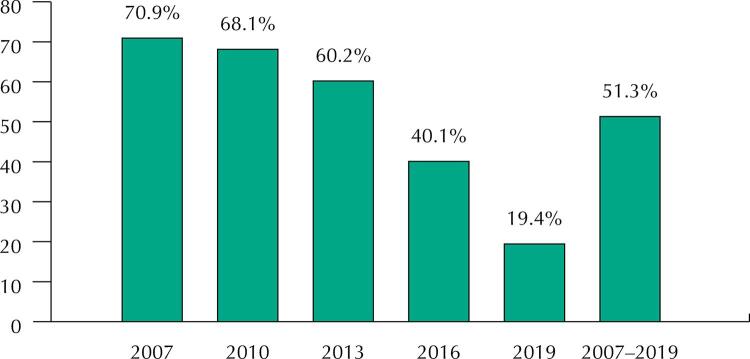
Occurrence of episiotomy between 2007 and 2019. Rio Grande, RS, Brazil. (n = 5,714).

The adjusted analysis showed a prevalence ratio (PR) for episiotomy among adolescent mothers of (PR = 2.23; 95%CI: 1.89–2.63) in comparison to those aged 35+ years. For mothers with nine years or more of schooling and in the highest income quartile, the PR were of (PR =1.21; 95%CI; 1.03–1.42) and (PR =1.25; 95%CI: 1.10–1.41) in comparison to those with 0–4 years of schooling and in lowest income quartile, respectively. Primiparous mothers showed (PR = 3.41; 95%CI: 2.95–3.95) of undergoing episiotomy in comparison to those with three or more children. The PR for those who delivered with a private sector and received oxytocin was (PR = 1.25; 95%CI: 1.07–1.46) and (PR = 1.18; 95%CI: 1.09–1.27), respectively. Among those who gave birth with the use of a forceps and had a child weighing over 4,000, PR for undergoing episiotomy were (PR = 1.32; 95%CI: 1.16–1.50) and (PR =1.43; 95%CI: 1.14–1.80), compared to those not submitted to forceps and whose child was born with low birth weight, respectively.

## DISCUSSION

Although episiotomy rate fell 3.6 times between 2007–2019, half of the puerperae underwent episiotomy in the studied period. The factors significantly associated with its occurrence were younger age, higher schooling, high household income, primiparous, attended by a private doctor during delivery, oxytocin use, forceps, and had a baby with a birth weight of at least 4,000 grams in this pregnancy.

The prevalence of episiotomy varies significantly between types of services (public and private) and locations^4,15^. Despite the maximum acceptable rate by the WHO of up to 10%^16^, reported rates of episiotomy at national level range from 9% in Sweden to 100% in Taiwan. This difference can be attributed to local policies regarding selective or routine use^4^.

However, in the last decades, episiotomy has markedly dropped at a global level^4,15^. In the U.S., within a cohort of 2,261.070 women who were hospitalized for a vaginal delivery in 510 hospitals, 325,193 underwent episiotomy (14.4%). There was a decline in rates of episiotomy; from 17.3%, in 2006, to 11.6%, in 2012^17^. In France, the rates decreased from 22%, in 2013, to 14%, in 2017^18^; while in Finland, this rate decreased from 71% to 55% among primiparous women, and from 21% to 9% among multiparous women, between 1997 and 2007^19^. The main reason behind this trend is that episiotomy began to be selective and not a routine procedure in many countries^2^. In France, the factors associated with episiotomy have not changed, suggesting that the decrease in episiotomies is probably due to proactive changes in its restrictive practices^20^.

No historical data about episiotomy in Brazil is available at the national level. A study named “*Nascer no Brasil”* (Born in Brazil), conducted in 2012–2013, found a nationwide rate of 56%, ranging from 49% in the North Region to 69% in the Midwest, and from 55% to 67% in public and private sectors^21^. This high nationwide rate is due to childbirth being performed mainly by doctors, whose practice is characterized by the excessive use of obstetric interventions, of which episiotomy persists as a routine procedure in many private hospitals^22^. The drastic reduction observed in the episiotomy rate in Rio Grande, from 71% in 2007 to 19% in 2019, may be related to a greater nursing participation, the presence of a companion in the pre-delivery period, and the current obligation by the Brazilian Ministry of Health for doctors to seek the women’s authorization to carry out this procedure^23^. Also, the partial closure of the hospital that served all patients from the private sector, where episiotomy was higher, could undoubtedly have contributed to this very sharp decline. Furthermore, the documentation of the procedures and the need for the mothers’ consent can inhibit the offering of unnecessary care or obstetric malpractice^23^ which may be occurring in Rio Grande.

The lower the age of the puerperae, the greater the probability of episiotomy. Among adolescents, for example, the (PR = 2.23; 95%CI: 1.89–2.63) compared to those aged 35 years or older. This higher occurrence is due to the lack of biological maturity, the muscles of pregnant adolescents have shown to be more tense than of older women, hindering the cephalic pole passage through the vaginal canal^1,4^; thus episiotomy is practiced in order to prevent perineal lacerations that would compromise vaginal delivery later on^1,10^.

Schooling and household income were also significantly associated with episiotomy among the studied women of Rio Grande. The dose-response effect clearly shows that the higher the schooling and income, the greater the likelihood of women having an episiotomy. The PR for those with nine years of schooling or higher reached (PR = 1.21; 95%CI: 1.03–1.42) compared to women with up to four years of schooling; mothers belonging to households of the higher quartile were 25% more likely to undergo episiotomy than those of the lowest quartile. This was also found in other settings^2,18^. It is noteworthy that these women are, knowingly, at lower risk of complications during childbirth, and yet they are more likely to undergo a procedure intended for those at greater risk; in Brazil, the same pattern is observed concerning C-sections, which is more common among the wealthiest women^24^.

The lower the number of children, the greater the likelihood to be submitted to episiotomy. Among the primiparous women, the episiotomy rate was 71% versus 22% among those who had three or more children – 3.3 times more – (Table 2). In regard to the effect measure, first births showed a (PR = 3.41; 95%CI: 2.95–3.95) compared to those with three or more children. Similar results were found in other settings^25^. The indication of episiotomy between primiparous could be related to the lower elasticity of perineal muscles^1,4,5^. Conversely, there will be an almost compulsory need to repeat the episiotomy in subsequent deliveries due to the greater fragility of the perineal musculature from the previous episiotomy, establishing a vicious circle^4,10^.

**Table 2:** Crude and adjusted analysis for factors associated with episiotomy. Rio Grande, RS, Brazil, 2007–2019.

Level	Variable	Prevalence ratio (95%CI)	

		Crude	Adjusted
I	Mother’s age (full years)	p < 0.001^a^	p < 0.001^a^
	11–19	2.09 (1.78–2.45)	2.23 (1.89–2.63)
	20–24	1.66 (1.41–1.95)	1.68 (1.42–1.97)
	25–29	1.55 (1.31–1.82)	1.54 (1.30–1.82)
	30–34	1.36 (1.14–1.62)	1.34 (1.12–1.61)
	≥ 35	1.00	1.00
	Skin color	p < 0.001	p = 0.419
	White	1.13 (1.01–1.27)	0.96 (0.89–1.05)
	Brown	1.10 (0.96–1.25)	0.93 (0.82–1.05)
	Black	1.00	1.00
	Mother’s schooling (full years)	p < 0.001^a^	p = 0.001^a^
	0 to 4	1.00	1.00
	5 to 8	1.20 (1.04–1.40)	1.04 (0.89–1.22)
	≥ 9	1.36 (1.18–1.58)	1.21 (1.03–1.42)
	Household monthly income (quartiles)	p < 0.001^a^	p = 0.001^a^
	First (lowest)	1.00	1.00
	Second	1.01 (0.92–1.12)	1.02 (0.92–1.12)
	Third	1.14 (1.04–1.26)	1.17 (1.05–1.29)
	Quarter (highest)	1.18 (1.06–1.33)	1.25 (1.10–1.41)
II	Parity	p < 0.001^a^	p < 0.001^a^
	1	3.26 (2.89–3.68)	3.41 (2.95–3.95)
	2	2.34 (2.07–2.66)	2.40 (2.10–2.76)
	≥ 3	1.00	1.00
III	Performed antenatal care visits	p < 0.001	p = 0.227
	Public sector	1.00	1.00
	Private sector	1.22 (1.12–1.32)	1.06 (0.96–1.18)
	Trimester of the first antenatal visit (n = 5,355)	p < 0.001^a^	p = 0.207
	First	1.51 (1.17–1.95)	1.25 (0.95–1.65)
	Second	1.30 (1.00–1.69)	1.18 (0.90–1.55)
	Third	1.00	1.00
	Number of medical visits performed	p < 0.001	p = 0.957
	0–1	1.00	1.00
	2–5	1.52 (1.25–1.84)	1.04 (0.70–1.52)
	≥ 6	1.80 (1.50–2.15)	1.02 (0.69–1.51)
	Type of health services used for delivery:	p < 0.001	p = 0.004
	Public	1.00	1.00
	Private	1.43 (1.25–1.64)	1.25 (1.07–1.46)
IV	Induction of labor	p < 0.001	p < 0.001
	Yes	1.29 (1.20–1.38)	1.18 (1.09–1.27)
	No	1.00	1.00
	Use of forceps	p < 0.001	p = 0.009
	Yes	1.68 (1.48–1.90)	1.32 (1.16–1.50)
	No	1.00	1.00
	Birth weight (in grams) (n = 5,706)	p < 0.001^a^	p = 0.001^a^
	< 2,500	1.00	1.00
	2,500–2,999	1.35 (1.16–1.56)	1.29 (1.11–1.50)
	3,000–3,499	1.47 (1.28–1.69)	1.38 (1.19–1.59)
	3,500–3,999	1.38 (1.18–1.60)	1.40 (1.20–1.63)
	≥ 4,000	1.29 (1.04–1.61)	1.43 (1.14–1.80)

^a^ Wald test for heterogeneity.

Delivery with a private doctor showed (PR = 1.25; 95%CI: 1.07–1.46) concerning those performed in the public sector. A retrospective cohort study conducted in Ireland with 403,642 women detected that women with private health insurance are at a higher risk of having an instrumental birth (RR = 1.25; 95%CI: 1.22–1.27) or an episiotomy (RR = 1.40; 95%CI: 1.38–1.43) in comparison to those who give birth in public hospitals^26^. Similar results were also found in New South Wales, in Spain, and in the Northeastern region in Brazil^27^. The cause of this higher rate of episiotomies may be due to the fact that there are more vaginal births in public hospitals than in private ones, where there is a higher rate of cesarean sections. In this scenario, episiotomy became a widespread practice, mainly in the private sector.

The PR for episiotomy between those who had received oxytocin previously was (PR = 1.18; 95%CI:1.09–1,27) compared to the others. This can also be the result of the excessive obstetric intervention in Brazil. In this case, oxytocin use can be just the starting point of a process ending with episiotomy^22,24^.

The use of forceps showed a (PR = 1.32; 95%CI: 1.16–1.50) for episiotomy. This combination increases the probability of severe perineal laceration and appears to be associated with fecal incontinence later^30^.

Finally, the greater the birth weight, the greater the likelihood of having an episiotomy. New mothers whose child was born with at least 4,000 grams showed a (PR = 1.43; 95%CI: 1.14–1.80) of undergoing episiotomy in comparison to those whose child had a low birth weight (< 2,500 grams). The greater the birth weight, the greater the baby’s difficulty of passing through the birth canal and; increasing the probability of cephalopelvic disproportion and of prolonged second stage of labor^30^. In this situation, episiotomy was the option.

Although there are indications for episiotomy, this procedure has been performed more than necessary in Rio Grande, facilitating lacerations, dyspareunia, and fecal and urinary incontinence^10^. This intervention must be urgently discontinued as a routine practice, or else it will cause more harm than good to the patient^4,10^. A parsimonious and selective practice should be implemented.

In the case of Rio Grande, it seems appropriate for us to further investigate the possible effects factors, such as the mandatory authorization by the puerperae, the presence of a family member in pre-delivery, a continuous care, and a nurse in the immediate pre-delivery, may have in the practice of episiotomy. These measures have possibly contributed to the drastic reduction of episiotomy observed in this municipality over the period studied. If this is confirmed, these measures could be reinforced among the professionals working in the immediate pre-delivery in all of Brazilian municipalities.
